# Strengthening Fungal Infection Diagnosis and Treatment: An In-depth Analysis of Capabilities in Honduras

**DOI:** 10.1093/ofid/ofae578

**Published:** 2024-10-03

**Authors:** Bryan Ortiz, Diana Varela, Gustavo Fontecha, Karla Torres, Oliver A Cornely, Jon Salmanton-García

**Affiliations:** Instituto de Investigaciones en Microbiología, Facultad de Ciencias, Universidad Nacional Autónoma de Honduras, Tegucigalpa, Honduras; Servicio de Infectología, Servicio de Atención Integral de Pacientes con VIH, Hospital Escuela, Tegucigalpa, Honduras; Instituto de Enfermedades Infecciosas y Parasitarias Antonio Vidal, Tegucigalpa, Honduras; Instituto de Investigaciones en Microbiología, Facultad de Ciencias, Universidad Nacional Autónoma de Honduras, Tegucigalpa, Honduras; Agrupación de Microbiólogos Propietarios de Laboratorios Privados de Honduras, Tegucigalpa, Honduras; Departamento de Química y Biología, Centro Universitario Regional de Occidente, Santa Rosa de Copán, Honduras; Faculty of Medicine and University Hospital Cologne, Institute of Translational Research, Cologne Excellence Cluster on Cellular Stress Responses in Aging-Associated Diseases, University of Cologne, Cologne, Germany; Faculty of Medicine and University Hospital Cologne, Department I of Internal Medicine, Center for Integrated Oncology Aachen Bonn Cologne Duesseldorf and Excellence Center for Medical Mycology, University of Cologne, Cologne, Germany; German Centre for Infection Research, Partner Site Bonn-Cologne, Cologne, Germany; Faculty of Medicine and University Hospital Cologne, Clinical Trials Centre Cologne, University of Cologne, Cologne, Germany; Faculty of Medicine and University Hospital Cologne, Institute of Translational Research, Cologne Excellence Cluster on Cellular Stress Responses in Aging-Associated Diseases, University of Cologne, Cologne, Germany; Faculty of Medicine and University Hospital Cologne, Department I of Internal Medicine, Center for Integrated Oncology Aachen Bonn Cologne Duesseldorf and Excellence Center for Medical Mycology, University of Cologne, Cologne, Germany; German Centre for Infection Research, Partner Site Bonn-Cologne, Cologne, Germany

**Keywords:** antifungal therapies, capacity building, diagnostic capabilities, health care infrastructure, invasive fungal infections

## Abstract

**Background:**

Invasive fungal infections (IFIs) are a major public health concern in low- and middle-income countries (LMICs) due to limited diagnostic and treatment resources, leading to high morbidity and mortality. Despite their significant global burden, IFIs are underrecognized and underdiagnosed in LMICs. This study evaluates the diagnostic and therapeutic capacities for managing IFI in Honduras, a country with unique health care challenges.

**Methods:**

From March to December 2023, a comprehensive survey was conducted across multiple health care centers in Honduras. The survey, reviewed for content and clarity by local medical institutions, targeted medical microbiologists and clinicians to assess various aspects of fungal disease diagnosis and treatment. Data included the availability and use of diagnostic tools and antifungal therapies, identifying gaps and limitations in current practices.

**Results:**

The survey revealed that *Candida* spp (97.4%) and *Aspergillus* spp (35.9%) were the most concerning pathogens. Although microscopy and culture methods were available in most institutions, their application in suspected IFI cases was inconsistent, and antifungal susceptibility testing was rarely performed. Advanced diagnostic techniques, such as antigen detection, were available in only a few institutions, while antibody detection and polymerase chain reaction testing were entirely absent. All hospitals had access to at least 1 triazole antifungal, typically fluconazole, but there was a notable scarcity of more potent antifungals, including amphotericin B formulations and echinocandins. The limited use of available diagnostic tools and the restricted availability of essential antifungals were identified as major barriers to effective IFI management.

**Conclusions:**

This study highlights significant gaps in the diagnostic and therapeutic capabilities for managing IFI in Honduras. The underutilization of basic diagnostic tools, the inaccessibility of advanced testing methods, and the limited availability of essential antifungal medications underscore the urgent need for capacity-building initiatives, infrastructure improvements, and policy reforms. Addressing these deficiencies is critical for enhancing the management of IFI in Honduras, with broader implications for similar LMIC settings. These findings can inform targeted interventions and resource allocation to improve outcomes for patients with IFI.

Invasive fungal infections (IFIs) pose a significant threat to public health, especially in low- and middle-income countries, where diagnostic and treatment capabilities are often limited [1–4]. IFIs are associated with high morbidity and mortality rates, particularly among patients who are immunocompromised, including those with HIV/AIDS, cancer, and organ transplants [5]. Despite the global burden of fungal diseases [1], they frequently remain underrecognized and underdiagnosed in low- and middle-income countries, primarily due to limited access to advanced diagnostic tools and antifungal treatments [6, 7].

Honduras is located in the center of the Central American isthmus (15°00′N 86°30′W). The country covers an area of 112 777 km^2^ and has a population of >9 million people [8], with 55.5% residing in rural areas and 44.5% in urban zones [9]. The Honduran health care system is divided into 2 sectors: public and private. The public sector consists of the Ministry of Health, which oversees regulation, direction, and provision of health services for the entire population, and the Honduran Institute of Social Security, which handles the collection and management of fiscal resources as well as mandatory contributions from workers and employers [10, 11]. The private sector includes for-profit and nonprofit health service providers [11, 12]. Currently, the Ministry of Health is responsible for medical care for at least 60% of the Honduran population, while the Honduran Institute of Social Security covers approximately 12% of the population. The private sector serves around 10% of the population. Consequently, 9 of 10 people in Honduras lack any form of health insurance, and an estimated 18% of the population, equivalent to >1.5 million Hondurans, has no access to any health services [10, 12]. The burden of severe fungal infections in Honduras is high, with an estimated 5% of the population at risk for IFI [13]. Thus, Honduras faces unique challenges in managing IFI [13]. The health care system in Honduras is characterized by disparities between urban and rural areas and between public and private ownership, with significant differences in the availability of medical resources and expertise [10, 13, 14]. Previous studies have highlighted the need for improved diagnostic capabilities and access to essential antifungal therapies in the region [13, 15]. However, there is a paucity of comprehensive data on the current state of fungal disease diagnosis and treatment across different health care settings in Honduras.

This study aims to fill this gap by providing a detailed assessment of the diagnostic and therapeutic capabilities for managing IFI in Honduras. This information is crucial for identifying areas that require intervention and for guiding future efforts to enhance the management of IFI in Honduras. The insights gained from this study can inform policy decisions, guide resource allocation, and support capacity-building initiatives aimed at enhancing the health care response to IFI in Honduras and similar settings.

## METHODS

**Figure 1.**
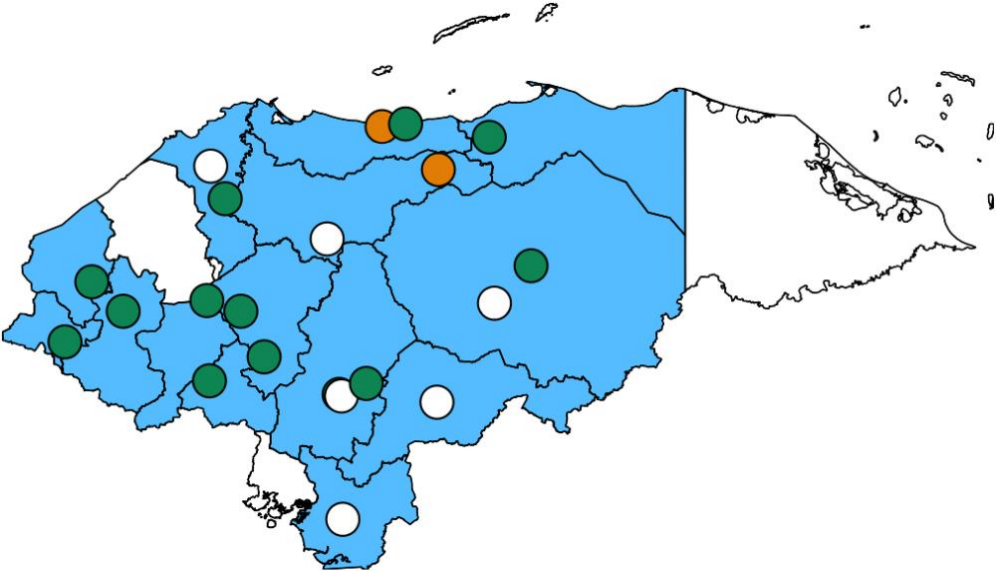
Map of participating institutions per “Departamentos” (regions). Departamentos with participating institutions are marked in blue, while those without are shown in white. If multiple centers are located in the same area, they are represented by a single circle. Circle colors indicate response sources: green for clinicians and microbiologists, white for microbiologists only, and orange for clinicians only.

A comprehensive study was carried out across multiple centers to assess the capabilities for diagnosing and treating IFI in Honduras (Figure 1). This involved the creation of a survey that examined various facets of fungal disease diagnosis and treatment (Supplementary Appendix). The survey, which ran from March to December 2023, targeted medical microbiologists and clinicians.

Before the survey was distributed, it underwent a review for content and clarity by staff from the Microbiology Research Institute, the Group of Microbiologists Owners of Private Laboratories of Honduras, the Antonio Vidal Institute of Infectious Diseases and Parasitology, and the Honduran Society of Infectious Diseases.

Upon approval, the survey was made accessible online and was accompanied by a video that explained its purpose and scope. The access link was disseminated via telephone and email to those affiliated with the participating institutions. Participation was voluntary and no incentives were offered.

Feedback was gathered from microbiologists on several aspects, including the nature of their laboratory (public or private), their perceptions of the prevalence and importance of IFI within their institution, the utilization of microscopy techniques, and the processes of fungal culture and identification. They also provided information on the availability of serologic, antigenic, and molecular tests. Clinicians similarly shared their views on the occurrence and significance of IFI within their institution and the accessibility of antifungal treatments and therapeutic drug monitoring.

Prior to the final exploratory analysis, each participant's responses were verified to ensure consistency and integrity. The data were summarized with SPSS version 27.0 (IBM Corp) and are presented as frequencies and percentages. Due to the uniformity of responses from participating institutions, no comparisons were made.

## RESULTS

This study recorded participation from centers in 14 of 18 departments, with at least 1 center from each participating department. Multiple laboratories took part in the survey in the departments of Francisco Morazán (n = 14, 35.9%), Comayagua (n = 5, 12.8%), Cortés (n = 4, 10.3%), and Olancho and Yoro (each n = 2, 5.1%). These departments collectively represent nearly 50% of the Honduran population. In contrast, no responses were received from Islas de la Bahía, Gracias a Dios, Valle, and Santa Bárbara. Except for Santa Bárbara, these departments are among the least populated in Honduras, accounting for only 3% of the country's population.

The participating clinics and hospitals were distributed across 7 departments, including the 2 main public specialty hospitals in the country, located in San Pedro Sula and Tegucigalpa. The distribution of participating laboratories and hospitals by department and city is detailed in the supplementary appendix.

### Access to Diagnostic Tools

Our survey encompassed 39 institutions, predominantly private laboratories (n = 34, 87.2%; Figure 1). The estimated incidence of IFI was very low in the majority of these institutions (n = 24, 61.5%), with a smaller proportion reporting low (n = 6, 15.4%) or moderate (n = 7, 17.9%; Table 1).

**Table 1.** Access to Laboratory Tools of Participating Institutions in Honduras

	No.	%
**Type of institution**		
Public laboratory	3	7.7
Public-private laboratory	2	5.1
Private laboratory	34	87.2
**Estimated IFI incidence**		
Very low	24	61.5
Low	6	15.4
Moderate	7	17.9
High	1	2.6
Very high	1	2.6
**Microscopy**	37	94.9
KOH	36	92.3
Chinese ink	14	35.9
Fresh examination	26	66.7
Lactophenol cotton blue stain	13	33.3
Gram stain	14	35.9
Giemsa stain	7	17.9
Fluorescence dyes	1	2.6
Silver staining in pneumocystosis suspicion	1	2.6
**Microscopy use if IFI suspected**		
Never	26	66.7
Almost never	9	23.1
Sometimes	3	7.7
Almost always	0	0.0
Always	1	2.6
**Culture**	38	97.4
Blood culture if fungemia suspected	3	7.7
Available media for fungal culture		
Chromogenic agar	9	23.1
Sabouraud	21	53.8
Sabouraud + chloramphenicol	3	7.7
Sabouraud + gentamicin	2	5.1
Sabouraud + chloramphenicol + cycloheximide	1	2.6
Available tests for species identification		
Molds		
Morphologic characterization	7	17.9
Yeasts		
Germ tube	23	59.0
Chromogenic medium	9	23.1
Manual biochemical methods	2	5.1
Automated identification system	4	10.3
Morphologic characterization	7	17.9
Antifungal susceptibility		
For yeast	2	5.1
Sensititre YeastOne	1	2.6
VITEK 2	2	5.1
None	37	94.9
**Antigen detection**	4	10.3
*Aspergillus* spp galactomannan: ELISA	1	2.6
Outsourced	1	2.6
*Candida* spp	1	2.6
Outsourced	1	2.6
*Cryptococcus* spp	3	7.7
LFA	3	7.7
In-house	2	5.1
Outsourced	1	2.6
LAT	3	7.7
In-house	3	7.7
*Histoplasma* spp	2	5.1
Outsourced	2	5.1

These answers were collected from microbiologists.

Abbreviations: ELISA, enzyme-linked immunosorbent assay; IFI, invasive fungal infection; KOH, potassium hydroxide; LAT, latex agglutination test; LFA, lateral flow assay.

**Figure 2.**
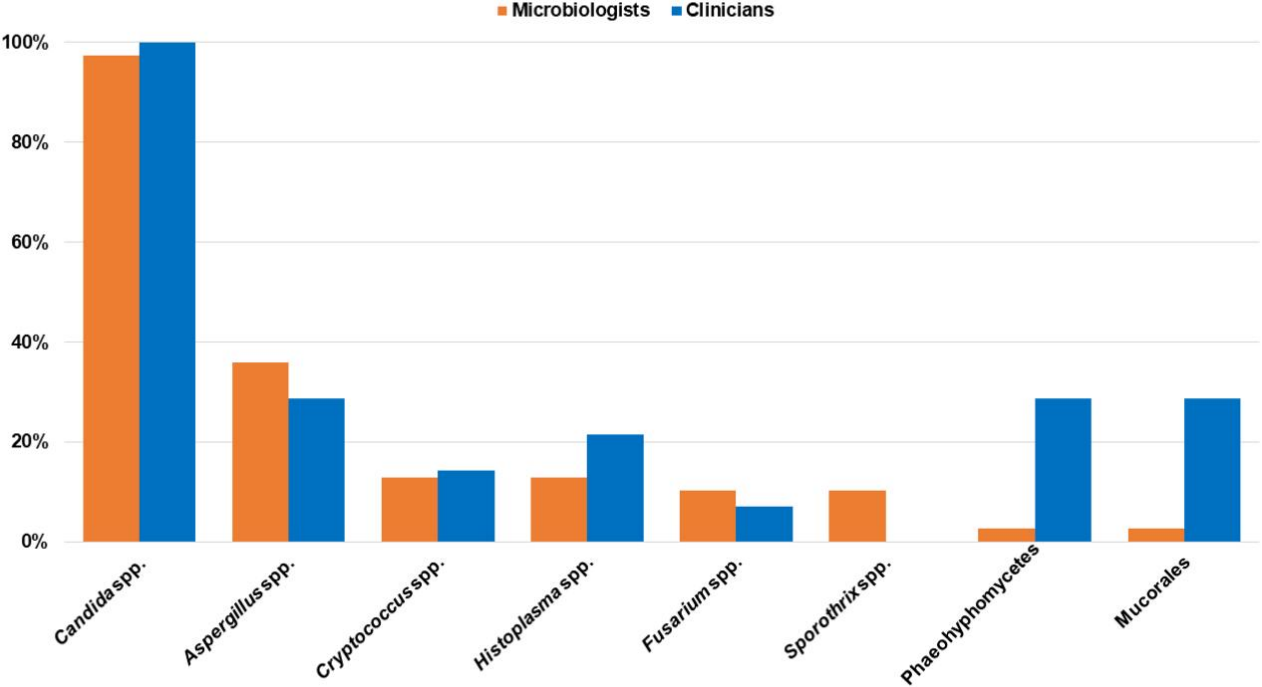
Fungal pathogens described as having the most relevance in Honduras.

In terms of pathogens, *Candida* spp (n = 38, 97.4%) and *Aspergillus* spp (n = 14, 35.9%) were estimated as the most prevalent within the institutions. Other pathogens were reported, such as *Cryptococcus* spp (n = 5, 12.8%), *Histoplasma* spp (n = 5, 12.8%), *Fusarium* spp (n = 4, 10.3%), and *Sporothrix* spp (n = 4, 10.3%), while no institutions identified *Coccidioides* spp, *Lomentospora*/*Scedosporium* spp, or *Paracoccidioides* spp as relevant pathogens (Figure 2).

Microscopy could be utilized in most institutions (n = 37, 94.9%), with potassium hydroxide (n = 36, 92.3%) and fresh examination (n = 26, 66.7%) being the most widely available methods. Other techniques included Chinese ink (n = 14, 35.9%), Gram stain (n = 14, 35.9%), lactophenol cotton blue stain (n = 13, 33.3%), and Giemsa stain (n = 7, 17.9%). However, in the context of suspected IFI, the use of microscopy varied, with most institutions (n = 26, 66.7%) never using it and only 1 institution (2.6%) reporting that it always used microscopy in these cases (Table 1).

Culture was available in almost all institutions (n = 38, 97.4%), with blood culture being performed in a few institutions if fungemia was suspected (n = 3, 7.7%). Sabouraud agar (n = 21, 53.8%) was the most commonly available medium for fungal culture, with 6 laboratories (15.3%) reporting the use of Sabouraud supplemented with antibiotics. Among these, 3 laboratories (7.7%) used Sabouraud with chloramphenicol, 2 (5.1%) used Sabouraud with gentamicin, and only 1 (2.6%) reported using Sabouraud supplemented with both antibiotics. Chromogenic agar was the second-most common medium, used by 6 laboratories (15.4%). Antifungal susceptibility testing was performed for yeasts in a reduced number of institutions (n = 2, 5.1%), with SensiTitre YeastOne and VITEK 2. However, no institutions performed this testing for molds (Table 1).

A limited number of institutions (n = 4, 10.3%) performed antigen detection, targeting mainly *Cryptococcus* spp (n = 3, 7.7%) and *Aspergillus* spp (n = 1, 2.6%). None of the institutions could perform antibody detection or polymerase chain reaction (Table 1).

### Access to Treatment Tools

Participants from 14 clinical wards provided estimates of IFI incidence within their units (Figure 1), ranging from very low (n = 7, 50.0%) to moderate (n = 3, 21.4%; Table 2). *Candida* spp (n = 14, 100.0%) emerged as the most prevalent pathogen, identified in all wards. *Aspergillus* spp, Mucorales, and phaeohyphomycoses were also encountered, albeit at a lower frequency (each n = 4, 28.6%; Figure 2). Radiograph and ultrasound (each n = 11, 78.6%) constituted the most frequently employed diagnostic imaging procedures.

**Table 2.** Access to Laboratory Tools of Participating Institutions in Honduras

	No.	%
**Estimated IFI incidence**		
Very low	7	50.0
Low	4	28.6
Moderate	3	21.4
High	0	0.0
Very high	0	0.0
**Imaging procedures**		
CT	6	42.9
MRI	4	28.6
Ultrasound	11	78.6
Radiograph	11	78.6
Bronchoscopy	7	50.0
Colonoscopy	8	57.1
Gastroscopy	8	57.1
Laryngoscopy	9	64.3
Nasal endoscopy	9	64.3
**Available antifungals**		
Amphotericin B, any	7	50.0
Deoxycholate	7	50.0
Lipid complex	0	0.0
Liposomal	3	21.4
Echinocandins	5	35.7
Anidulafungin	3	21.4
Caspofungin	2	14.3
Micafungin	0	0.0
Triazoles	14	100.0
Fluconazole	13	92.9
Isavuconazole	0	0.0
TDM	0	0.0
Itraconazole	6	42.9
TDM	0	0.0
Posaconazole	0	0.0
TDM	0	0.0
Voriconazole	5	35.7
TDM	0	0.0
Flucytosine	0	0.0
TDM	0	0.0
Terbinafine	4	28.6
**Surgery**	6	42.9

These answers were provided by clinicians.

Abbreviations: CT, computed tomography; IFI, invasive fungal infection; MRI, magnetic resonance imaging; TDM, therapeutic drug monitoring.

In regard to antifungal therapy, all participating hospitals possessed access to at least 1 triazole (n = 14, 100.0%). Fluconazole (n = 13, 92.9%) was the most widely available triazole, while itraconazole (n = 6, 42.9%) was the most common mold-active agent within this class. The capacity to administer amphotericin B (n = 7, 50.0%), mainly deoxycholate formulation (n = 7, 50.0%), was present in half of the surveyed institutions. Conversely, access to echinocandins proved to be more restricted, with only 35.7% (n = 5) of wards reporting availability. Flucytosine was not accessible in any of the participating institutions. Surgical intervention for IFI management remained an option in 42.9% (n = 6) of the surveyed wards (Table 2).

## DISCUSSION

Our study surveyed 39 clinical wards and microbiological laboratories across health care institutions in Honduras. While most institutions have access to microscopy and culture methods, their use in suspected IFI cases is limited. Antifungal susceptibility testing is rare and primarily available for yeasts. Antigen detection is performed in a small number of institutions, while antibody detection and polymerase chain reaction are unavailable nationwide. Diagnostic imaging, such as radiographs and ultrasounds, is commonly used. All hospitals have access to at least 1 triazole, mainly fluconazole, but only half can administer amphotericin B, and echinocandins are also limited. Flucytosine is not accessible, and surgical intervention for IFI is available in less than half of the wards.

A Pan-American initiative previously documented the therapeutic and diagnostic capabilities for fungal infections across 24 countries in Latin America and the Caribbean [4]. In that study, only 1 laboratory from central Honduras was evaluated, leaving Honduras underrepresented as compared with other countries. In contrast, our study gathered responses from 39 laboratories across 14 departments in the country to assess the diagnostic tools available in Honduras. Additionally, we received responses from 14 health care centers in 7 departments, focusing on the capacity to manage and access antifungal treatment. Notably, this includes participation from the country's main public specialty hospitals, where the most complex cases are typically treated. While the number of participating institutions may seem limited, we believe that the surveyed laboratories and hospitals represent a broad range of regions throughout Honduras. This approach ensures that the study highlights disparities in access and diagnostic capabilities that may exist among areas with varying levels of economic development, infrastructure, and medical resources. It provides an accurate reflection of the common realities regarding access to diagnosis and treatment of IFI in the country. Furthermore, this approach allows for the identification of patterns and trends that can be extrapolated to a broader context, making the study's findings relevant and valuable for national-level decision making.

When microbiologists and clinicians were consulted about the primary fungus associated with IFI in Honduras, *Candida* spp emerged as the most prominent pathogen, followed by *Aspergillus* spp and *Cryptococcus* spp. These pathogens align with the World Health Organization's list of priority fungal pathogens [16], and they are consistent with findings from other countries [2, 17–21]. Interestingly, microbiologists consider *Sporothrix* spp to be more relevant, while physicians emphasize the importance of phaeohyphomycetes and Mucorales, highlighting differences in exposure as well as clinical vs laboratory priorities. Our hypothesis is that microbiologists frequently encounter *Sporothrix* spp in laboratory diagnostics due to its specific culture requirements. Additionally, since sporotrichosis is primarily a cutaneous infection, patients infected with this fungus are often outpatients seen by general practitioners and dermatologists. These physicians tend to refer patients to any laboratory where these fungi can be cultured. Furthermore, the prevalence of this fungus in soil and plants in tropical regions is typically high [22], which could be significantly correlated with some cases reported in Honduras among patients involved in agriculture [23, 24]. This hypothesis gains validity considering that agriculture is one of the main economic activities in the country [25], correlating well with the literature that highlights the strong association of sporotrichosis with occupational infections related to agriculture [22]. Yet, infections caused by phaeohyphomycetes and Mucorales are typically severe, rapidly progressing infections that occur in patients who are immunocompromised. These cases require intervention by specialists, usually infectious disease physicians, who are generally restricted to tertiary care hospitals. This could explain the observed differences and why laboratories prioritize sporotrichosis over mucormycosis. In any scenario, geographic and environmental factors, along with epidemiologic reporting practices, may influence these differing perspectives, emphasizing the importance of personalized approaches in managing IFI. Additionally, there is an urgent need to improve case reporting practices in the country, which would contribute to a better understanding of these mycoses [24, 26, 27].

Previous researchers conducted a comprehensive analysis of scientific evidence to determine the burden of IFI in Honduras. Our findings support their conclusions, indicating that microscopic examination and cultures remain the primary methods of laboratory diagnosis in the country [13]. However, microscopy and culture typically exhibit limited sensitivity in isolating and identifying fungi, frequently depending on the sample used. Given Honduras's restricted access to diagnostic services, this may be a factor in the low incidence that many institutions report [28–30], which may account for the perceived low-incidence perception of IFI reported by surveyed institutions.

When queried about available microscopy techniques, most laboratories reported using wet examination, potassium hydroxide slides, Gram stain, and Chinese ink. These techniques are valuable initial steps in diagnosing IFI. In an optimal laboratory setup, microscopy-based techniques offer crucial insights into microbial elements (eg, pseudo hyphae, septate hyphae, nonseptate hyphae, pigmented hyphae, and yeasts), especially from sterile sites, allowing clinicians to initiate antifungal therapy promptly [31], even before definitive culture results [31, 32]. Despite the widespread availability of microscopic techniques for fungi in most Honduran laboratories, their use is infrequent. This can be attributed to the absence of mandatory reporting requirements for IFI in Honduras and a lack of standardized procedures for handling samples. Laboratory algorithms for processing biological samples often rely on test requests from physicians, so without medical suspicion, laboratory technicians may limit their analysis to only requested tests. This highlights the need for standardized protocols to ensure comprehensive fungal examination, even without suspicion of infection.

Culture, despite its limitations, remains a reference technique for diagnosing IFI [28, 31, 33]. According to the European Organization for Research and Treatment of Cancer and the Mycoses Study Group Education and Research Consortium, isolating any fungus from a sterile site is considered a proven diagnosis of IFI [32]. Our findings are promising, as most laboratories confirm having solid media for fungal isolation. However, blood culture availability is limited as compared with other countries [17–21, 34], underscoring the need to enhance access to these tests in public and private settings.

Serologic tests based on antigen detection are invaluable due to their high sensitivity and specificity in diagnosing IFI such as aspergillosis, blastomycosis, coccidioidomycosis, cryptococcosis, histoplasmosis, and paracoccidioidomycosis [29, 35–41]. While serology-based techniques are available, they are not common among the surveyed laboratories. Honduras has made notable progress in researching and diagnosing cryptococcosis and histoplasmosis, derived from studies validating serologic tests for these infections in patients with HIV positivity in major health care centers [42, 43] . Given the high prevalence of IFI among a population with over half a million individuals experiencing illnesses and debilitating diseases [13], rapid diagnosis is a priority. Implementing these tests in health care centers is crucial. Although techniques in molecular biology and MALDI-TOF MS (matrix-assisted laser desorption ionization–time of flight mass spectrometry) are not available in surveyed laboratories, nucleic acid amplification tests have become popular in clinical settings over the last 2 decades, sometimes replacing traditional diagnostics [44]. Commercial techniques such as Filmarray, LightCycler SeptiFast, SepsiTest, and T2 *Candida* and in-house methods have shown promising results [28, 29, 45].

While no surveyed laboratories utilize molecular techniques for diagnosing IFI, the use of nucleic acid amplification techniques for fungal illnesses in Honduras is extremely limited and available only in private laboratories. The high cost of these tests, ranging from US $200 to $250, makes them unaffordable for most people, considering that the minimum monthly salary is around US $480 [46]. Sequencing techniques are primarily used in academic and research settings, with collaborative efforts proving insufficient [47, 48]. Additional support initiatives for implementing molecular biology–based methods would be beneficial. Likewise, MALDI-TOF MS–based techniques are unavailable in the country. Successful public-private strategies, such as those developed for malaria, tuberculosis, and HIV control, are necessary to ensure access to these tests for more people [49–52]. A good example of successful interventions for IFI in middle-income countries is the work done by the Global Action Fund for Fungal Infections in Guatemala [53, 54], a neighboring country of Honduras.

Regarding antifungal sensitivity tests, their availability is low and restricted to commercial tests for yeasts. Susceptibility tests for filamentous fungi are not performed in Honduras. The microdilution techniques recommended by the Clinical Laboratory Standards Institute and the European Committee on Antimicrobial Susceptibility Testing are also inaccessible in Honduras. Antifungal susceptibility testing is essential for guiding fungal disease treatment. The recent burden of IFI in Honduras, including the increase in mucormycosis associated with COVID-19 and reports of emerging and reemerging yeasts in the country [47, 55–57], justifies the need to implement these tests.

Overall, the lack of diagnostic tests for fungi can be attributed to several factors. The relatively small Honduran market may deter the industry from marketing and distributing these tests. Additionally, Honduras allocates one of the lowest amounts of funds to the health sector in the Americas [10, 12, 58], resulting in limited budgets for acquiring these tests in national hospitals, as well as delays in their supply, which means that their constant availability cannot be guaranteed.

In parallel, Honduras lacks a national reference laboratory for medical mycology that provides centralized diagnostic services in line with international standards. Establishing a well-equipped reference laboratory in medical mycology with highly skilled personnel would greatly benefit the country by providing diagnoses of severe fungal diseases and conducting antifungal sensitivity analyses. It could also contribute to the early detection of hospital outbreaks, epidemics, and possible pandemics. Models for implementing reference laboratories in low- and middle-income countries have been proposed and successfully implemented in some cases [59–61]. These examples offer valuable insights for managing and implementing a national reference laboratory for medical mycology in Honduras, focusing on the list of fungal pathogens proposed by the World Health Organization in 2022. These initiatives require a supportive political environment from decision makers.

While various diagnostic tools for medical mycology are available in Honduras, some remain underutilized. This underutilization may be related to the shortage of professionals and specialists in medical mycology, as well as the limited training opportunities in this field. One of the major challenges in advancing the understanding and improvement of IFI diagnosis is strengthening education in this area. Currently, only a few microbiology programs include a course dedicated to the study of fungi, while related health fields such as medicine, nursing, and dentistry cover this topic in only a few classes. There is an urgent need to reassess the importance of mycology in educational programs in Honduras and globally. By enhancing the curriculum to include more comprehensive training in mycology, we can ensure the development of more mycologists across various health disciplines, ultimately contributing to the timely diagnosis of fungal infections.

Access to antifungal treatments varied considerably. While all participating hospitals have at least 1 triazole antifungal, typically fluconazole, the availability of mold-active agents such as itraconazole and amphotericin B, especially liposomal formulation, is more limited. Echinocandins, as first-line therapy for invasive candidiasis [62–66], are available in about a third of the institutions, and flucytosine is not accessible at all. This limited availability is concerning given the importance of these drugs in treating IFI [37–39, 62–70]. For instance, combination therapy with flucytosine and liposomal amphotericin B is recommended for cryptococcal meningitis [37], a life-threatening condition prevalent among individuals infected with HIV. The disparity in antifungal availability highlights significant gaps in the treatment capabilities for IFI in Honduras. In high-resource settings, a broader range of antifungals is typically available, including liposomal formulations of amphotericin B, which are less nephrotoxic, and newer triazoles, such as posaconazole and isavuconazole, which have broader antifungal spectra. The absence of these critical medications in Honduras likely limits the ability to effectively manage complex IFI cases and could lead to higher mortality rates.

This study's limitations include the potential subjectivity introduced by relying on self-perceived incidence rates, which may not accurately reflect the actual incidence. Despite these limitations, this survey highlights areas for improving IFI diagnosis in Honduras. This multicenter study provides a comprehensive analysis of the diagnostic and therapeutic capacities for IFI in Honduras, revealing significant gaps and areas for improvement. These findings are crucial for understanding the current state of IFI management and guiding future interventions. The study also underscores the need for capacity-building initiatives to improve the diagnostic and therapeutic management of IFI. Training programs should focus on enhancing the skills of medical microbiologists and clinicians in identifying and managing IFI, including hands-on training in microscopy, culture techniques, and interpreting advanced diagnostic tests. Increasing awareness of the clinical significance of IFI among health care providers can help in early recognition and prompt treatment. Policy changes at the national and institutional levels are crucial to address the gaps identified in this study. Investment in health care infrastructure is needed to expand the availability of advanced diagnostic tools and essential antifungal medications. Government and health authorities should prioritize funding for procuring these resources and ensure their equitable distribution across health care facilities. International partnerships and collaborations can support these efforts by providing technical assistance, funding, and access to advanced diagnostics and treatments.

In conclusion, this study reveals significant gaps in the diagnostic and therapeutic capacities for IFI in Honduras. To improve IFI management in the region, it is essential to enhance access to advanced diagnostic tools and effective antifungal treatments, along with implementing targeted capacity-building initiatives. These findings align with broader trends seen in other low- and middle-income countries, highlighting the need for coordinated global efforts to tackle these disparities. Future research should focus on longitudinal studies to track progress and evaluate the impact of interventions designed to strengthen the health care response to IFI in Honduras. Moreover, addressing the academic aspects is crucial, such as cultivating a culture of case reporting and expanding continuous education programs and advanced degrees in the field. Improving infrastructure and access to better diagnostic methods also depends significantly on decision makers, who are often bureaucrats and politicians. Overcoming these challenges will require a collaborative effort from health care providers, policy makers, and international health organizations to ensure timely and effective care for patients with IFI.

## Supplementary Material

ofae578_Supplementary_Data

## Supplementary Data

Supplementary materials are available at *Open Forum Infectious Diseases* online. Consisting of data provided by the authors to benefit the reader, the posted materials are not copyedited and are the sole responsibility of the authors, so questions or comments should be addressed to the corresponding author.

## Notes

***Authorship.*** B. O. and J. S.-G. made substantial contributions to the study concept and design, data verification, and statistical analysis and interpretation of data and drafted the manuscript. All authors made substantial contributions to the acquisition of data for the work and critically reviewed the manuscript and gave final approval for publication.

***Collaborators.*** Susan Mejía Valladares, Fanny Yaneth Hernández Canales, Edwin Mejía, Sandra María Lara Orellana, Marisela Durón Contreras, Ana Samary Banegas Buezo, Doria Espinal, Luis Armando Velásquez Sosa, Dora Argentina Rodríguez, Mariela Medina, Erika Patricia Espinal Padilla, Leslie Jackeline Murillo Castillo, María Antonieta Molina, Karla Hernández, Carlos Ramón Hyde Castillo, Rafael Humberto Laínez Solorzano, Geovanna Padilla Durón, Adelaida Cruz, Sara Eloísa Rivera Molina, Sandra Lizeth Paredes Romero, Teresa Calix, Rosendo Alberto Contreras Gamero, Grezzia Fiallos, Gina Martínez, Oscar Gómez Madrid, Elena Mejía Arita, Wendi Vallecillo, Ingrid María Álvarez Flórez, Daniel Madrid, Rossana Granati Medina, Dania Merary Mayorquín Lanza, Karla Melissa Rico Rivas, Victoria Naira Sabillón, Xaman Méndez, Wendy Karely Moncada Navas, Verónica Pineda Downing, Annette Elizabeth Bendeck Sansur, Denise Jiménez Buchhalter, Carol M. Cruz Flores, Nery E. Linárez Ochoa, Mirna Leonor Molina, Pamela Zacasa, Georgina Celeste Galindo Morales, Linda Herrera, Carminda Noemí Sosa Montenegro, Ana Dolores Hernández Melara, Suyapa Euceda Pineda, Zila Belinda Turcios Ávila, Carmen Alicia Castillo, Ada Josefina Andrews Bulnes, Cinthia Reyes Almendárez, Eimy Barahona Moncada, Fany Gissela Rivera Alvarenga.

***Data sharing.*** Data will be shared upon reasonable request after contacting the corresponding author.

***Patient consent statement.*** Ethics approval and informed consent were not required per the guidelines for good clinical practice of the International Council for Harmonisation of Technical Requirements for Pharmaceuticals for Human Use, as this survey study included only responses by physicians without any patient-specific data. Ethics approval and informed consent are not mandatory for research not involving human subjects or biomaterials according to German law (§41a AMG).
