# Supplementary material for: Strengthening Fungal Infection Diagnosis and Treatment: An In-depth Analysis of Capabilities in Honduras
Source: Open Forum Infect Dis. 2024 Oct 3;11(10):ofae578. doi: 10.1093/ofid/ofae578 (PMC11483579; doi:10.1093/ofid/ofae578)
Supplement: ofae578_Supplementary_Data [file ofae578_supplementary_data.docx]

**APPENDIX**

**Appendix 1.** Data collected per Departamento (region)

|  | **Departamento** | **Location (n)** | **Answers per Departamento** |
| --- | --- | --- | --- |
| **Laboratory sites** | Atlántida | La Ceiba (1) | 1 |
|  | Choluteca | Choluteca (2) | 2 |
|  | Colón | Tocoa (1) | 1 |
|  | Comayagua | La Libertad (1)  Siguatepeque (2)  Comayagua (2) | 5 |
|  | Copán | Santa Rosa de Copán (1) | 1 |
|  | Cortés | San Pedro Sula (4) | 4 |
|  | El Paraíso | Danlí (2) | 2 |
|  | Francisco Morazán | Tegucigalpa (12)  Comayagüela (1)  Valle de Ángeles (1) | 14 |
|  | Intibucá | Jesús de Otoro (1) | 1 |
|  | La Paz | Marcala (1) | 1 |
|  | Lempira | Gracias (2) | 2 |
|  | Ocotepeque | San Marcos (1) | 1 |
|  | Olancho | Catacamas (1)  Juticalpa (1) | 2 |
|  | Yoro | Santa Rita (1)  Yorito (1) | 2 |
| **Clinical sites** | Atlántida | La Ceiba (1) | 1 |
|  | Choluteca | Choluteca (2) | 2 |
|  | Cortés | San Pedro Sula (1) | 1 |
|  | Francisco Morazán | Tegucigalpa (7) | 7 |
|  | El Paraíso | Danli (1) | 1 |
|  | Olancho | Juticalpa (1) | 1 |
|  | Yoro | Olanchito (1) | 1 |

**Appendix 2.** Survey used for data collection

**1. IFI Management Capacity Questionnaire**

Bienvenida/o al cuestionario online: "Evaluación de las Capacidades Diagnósticas y Acceso al Tratamiento Antifúngico en Honduras"

Este estudio es una iniciativa de la Universidad de Colonia (Colonia, Alemania), el Instituto de Investigaciones en Microbiología, de la Universidad Nacional Autónoma de Honduras (IIM-UNAH), el Laboratorio Nacional de Vigilancia de La Secretaría de Salud de Honduras (LNV-SESAL), la Agrupación de Microbiólogos Propietarios de Laboratorios Privados de Honduras (AMIPROLABPH), el Instituto de Enfermedades Infecciosas y Parasitología Antonio Vidal (IAV), la Sociedad Hondureña de Enfermedades Infecciosas (SHEI) y el Colegio Médico de Honduras (CMH).

En esta oportunidad nuestras instituciones de la mano de la Universidad de Colonia se encuentran comprometidos con el desarrollo y actualización de la Micología médica en el país, en este sentido hemos unido fuerzas para determinar las capacidades diagnósticas y el acceso al tratamiento antifúngico en Honduras.

Bajo este contexto, esta encuesta tiene como fin ayudar a comprender las prácticas actuales en los servicios de laboratorio de micología y las necesidades de avanzar en esta área del conocimiento, Agradecemos mucho su apoyo al responder a esta encuesta de la manera más completa posible. Estamos seguros de que la unión de nuestros esfuerzos ayudará a contribuir al avance de los servicios de diagnóstico de las micosis humanas en toda Honduras.

El estudio está coordinado por:

Dr. Bryan Ortiz (Honduras), bryan.ortiz@unah.edu.hn (IIM-UNAH)

Dra. Mitzi Castro (Honduras, jefaturalab2020@gmail.com (LNV-SESAL)

Dr. Jon Salmanton-García (Alemania), jon.salmanton-garcia@uk-koeln.de (UzK)

Dra. Karla Torres (Honduras), laboratoriosprivados@hotmail.com (AMIPROLABH)

Dra. Diana Varela (Honduras), ds_varela@hotmail.com (IAV/ SHEI/CMH)

Haciendo "click" en "CONTINUAR" acepto voluntariamente participar en este estudio, comprendo los procesos del proyecto, también entiendo que puedo decidir no participar y que puedo retirarme del estudio en cualquier momento, sin perjuicio alguno hacia mi persona.

**2. Información de contacto**

Nombre y apellidos

E-mail

Institución

Nombre institución, sección, etc.

Municipio

Departamento

Indique la sección del cuestionario en la cual quiere participar

Médicas/os - Manejo clínico y acceso al tratamiento de infecciones fúngicas en Honduras

Microbiólogas/os - Prácticas de laboratorio para el diagnóstico de infecciones fúngicas en Honduras

**2.1 Institution Profile**

Tipo de institución

Seleccione al menos una de las siguientes opciones.

Laboratorio público

Laboratorio público-privado

Laboratorio privado

Otro. Por favor especifica:

**2.2 Lab - Perceptions on invasive fungal disease in your institution**

Califique la incidencia de infecciones fúngicas invasivas en su institución

de muy bajo (1) a muy alto (5)

1

2

3

4

5

¿Cuáles son los hongos de mayor importancia en su centro en su opinión?

Marque todo lo que corresponda.

Aspergillus spp.

Candida spp.

Coccidioides spp.

Cryptococcus spp.

Fusarium spp.

Histoplasma spp.

Lomentospora/Scedosporium spp.

Paracoccidioides spp.

Sporothrix spp.

Feohifomicetos (hongos negros)

Mucorales

Otro. Indique por favor:

**2.3 Microscopy**

¿Qué metodologías utilizan en la microscopía de hongos?

Preparación de hidróxido de potasio (KOH)

Tinta china

Examen en fresco

Tinción azul de algodón de lactofenol

Tinción metenamina-plata de Grocott-Gomori

Tinción de Gram

Tinción de Giemsa

Otros (por favor especifique)

Ninguno, no disponemos ningún método para el diagnóstico micológico a nivel de microscopía

¿Con qué frecuencia realiza microscopía de muestras provenientes de sitios estériles o lavado broncoalveolar cuando sospecha de enfermedad fúngica?

de nunca (1) a siempre (5)

1

2

3

4

5

¿Tiene acceso a tintes de fluorescencia?

Sí

No

Cuando se sospecha criptococosis, ¿está disponible el examen directo en los fluidos corporales?

Sí, tinta china

Sí, otros tintes, (por favor especifique)

No

¿Cuando se sospecha pneumocistosis se realiza tinción de plata (metenamina-plata de Grocott-Gomori)?

Sí

No

Ante la sospecha de mucormicosis, ¿se realiza microscopía directa con blanqueadores ópticos (optical brighteners)?

Sí

No

En promedio, ¿cuánto tiempo lleva obtener el resultado de laboratorio en pacientes con sospecha de infección fúngica de…

Número de días

Microscopía

Histopatología incluyendo tinciones fúngicas

**2.4 Culture and Fungal Identfication**

¿Se dispone de hemocultivos automatizados ante la sospecha de fungemia?

Sí

No

Por favor marque todos los métodos usados para cultivos fúngicos

Agar semilla de girasol

Agar cromogénico para identificación de levaduras

Agar Lacrimel

Agar papa dextrosa

Sabourad

Sabouraud + Cloranfenicol

Sabouraud + Gentamicina

Sabouraud Cloranfenicol + cicloheximida

Otros (por favor especifique)

Ninguno de los anteriores

Seleccione todas las pruebas disponibles para la identificación de especies

Hongos filamentosos

Levaduras

Tubo germinal

Medio cromogénico

Métodos bioquímicos manuales

Kit de identificación semiautomatizado

Sistema de identificación automatizado

MALDI-TOF

Secuenciación de ADN

Caracterización morfológica

Otros (por favor especifique debajo)

Ninguno, no realizamos ninguna prueba interna para la identificación por especie

Pruebas disponibles para la identificación de especies (Otros)

¿Tiene acceso a pruebas de susceptibilidad a los antifúngicos?

Para mohos u hongos filamentosos

Para levaduras

Para ambos

Ninguno

¿Cuáles de las siguientes tecnologías para pruebas de susceptibilidad están disponibles?

ATB™ FUNGUS 3

Método de difusión por disco

Sensititre YeastOneTM

E-test

VITEK® 2

FUNGIFAST®

Método de microdilución usando estándares del CLSI

Método de microdilución usando estándares del EUCAST

Ninguno, no evaluamos susceptibilidad a antifúngicos

Elija la respuesta que mejor se adapte a la capacidad máxima de identificación en su laboratorio

Género

Género / especie

Género / especie / complejo

Género / especie / complejo / críptica

En promedio, ¿cuál es el tiempo de respuesta desde que se extrae la muestra hasta que se obtiene el resultado de una prueba microbiológica en pacientes con sospecha de infección fúngica de…

Número de días

Cultivo - hongos mohos o filamentosos

Cultivo – levaduras

**2.5 Serology**

¿Cuál de estas técnicas de diagnóstico serológico de enfermedades fúngica está a su disposición?

Aspergillus galatomanano (ensayo inmunoenzimático en microplacas tipo sándwich)

Aspergillus galactomanano (ensayo de flujo lateral)

Antígeno de Candida

Cryptococcus (ensayo de flujo lateral)

Cryptococcus (prueba de aglutinación de látex)

Histoplasma

Betaglucano (Prueba G)

Otros (por favor especifique debajo)

**2.6 Molecular Tests**

¿Dispone de alguna de las siguientes pruebas moleculares?

PCR para Aspergillus

PCR para Candida

PCR para Pneumocystis

PCR para mucorales

PCR para otros hongos, ¿Cuáles? (por favor especifique debajo)

¿Cuáles de las siguientes muestras pueden ser procesadas en su institución para pruebas moleculares?

Biopsias, punciones y secreciones de heridas profundas

Médula ósea, huesos

Muestras del área de ojos, oídos, nariz y garganta

Secciones de parafina

Materiales respiratorios, incluidos lavado broncoalveolar, hisopos respiratorios, esputo, secreciones traqueales, bronquiales y nasofaríngeas

Sangre

Ninguna de las anteriores

**3.1 Institution Profile**

Su cargo

Médica/o adjunto

Médica/o adjunto - Especialista en enfermedades infecciosas

Microbióloga/o clínica/o

Director/a

Médica/o especialista en control de infecciones

Profesional de laboratorio

Catedrática/o

Otra/o. Por favor, especifique:

Tipo de institución

Seleccione al menos una de las siguientes opciones.

Hospital o clínica de día

Clínica de diálisis

Instituto / Hospital de Investigación

Clínica de Oncología

Hospital privado

Laboratorio Privado

Hospital público

Hospital Universitario

Otro. Por favor especifica:

Tamaño de la institución - número de camas

Si no corresponde, ingrese "0".

Total

Camas UCI adultas/os

Camas UCI pediátrica/neonatal

¿Su institución atiende a pacientes con alguna de las siguientes condiciones?

Posible selección múltiple

COVID-19

Diabetes mellitus

Hematología

VIH/SIDA

Unidad de Cuidados Intensivos: Adultas/os

Unidad de Cuidados Intensivos: Neonatal

Oncología

Nutrición parenteral

Unidad de cuidados intensivos respiratorios/otros intermedios

Trasplante de órganos sólidos

Trasplante de células madre/hematopoyético

¿Cuenta su institución con un laboratorio de microbiología?

Sí, en la propia institución

Sí, externalizado a otra institución

No

¿Dónde se realizan los procedimientos diagnósticos micológicos?

Siempre en nuestra institución

Parte en nuestra institución / parte externalizada

Totalmente externalizada

No tenemos acceso a herramientas de diagnóstico micológico

¿Cuenta con un/a microbióloga/o especialista en micología que se encargue de las consultas?

Siempre en nuestra institución

Parte en nuestra institución / parte externalizada

Totalmente externalizada

No contamos con un/a especialista en micología médica responsable de las consultas

¿Cómo se informa a las/os médicas/os cuando el laboratorio detecta infecciones fúngicas invasivas?

Selección múltiple posible

Correo electrónico

Sistema de información de laboratorio

Beeper / Buscapersonas

Llamada telefónica

Mensaje de texto/SMS

Otro. Por favor, especifique:

¿Consulta regularmente a un laboratorio de referencia?

No

Sí. Por favor, especifique cuántas veces al año aproximadamente:

Desconocido

**3.1.1 Prevalencia**

Califique la incidencia de infecciones fúngicas invasivas en su institución

de muy bajo (1) a muy alto (5)

1

2

3

4

5

¿Cuáles son los hongos de mayor importancia en su centro en su opinión?

Marque todo lo que corresponda.

Aspergillus spp.

Candida spp.

Coccidioides spp.

Cryptococcus spp.

Fusarium spp.

Histoplasma spp.

Lomentospora/Scedosporium spp.

Paracoccidioides spp.

Sporothrix spp.

Feohifomicetos (hongos negros)

Mucorales

Otro. Indique por favor:

Indique todos los medicamentos disponibles para el tratamiento antimicótico en su institución

Por favor conteste cada pregunta. En caso de que falte información, seleccione "desconocido".

Anfotericina B desoxicolato

Anfotericina B complejo lipídico

Anfotericina B liposomal

Anfotericina B - otras formulaciones

Anidulafungina

Caspofungina

Fluconazol

Flucitosina (5-FC)

Isavuconazol

Itraconazol

Micafungina

Posaconazol

Terbinafina

Voriconazol

Otro. Por favor, indique:

**3.1.2 Monitoreo**

¿Su institución tiene acceso a monitoreo de la concentración sérica de medicamentos terapéuticos de agentes antifúngicos?

Por favor conteste cada pregunta. En caso de que falte información, seleccione "desconocido".

5-flucitosina

Isavuconazol

Itraconazol

Posaconazol

Voriconazol

**3.1.3 Diagnóstico por imagen**

Por favor, seleccione cuál de los siguientes procedimientos de imagen está disponible en su institución para el diagnóstico de infecciones fúngicas invasivas

° “Intervencional” significa cualquiera de aquellas endoscopias que tienen como objetivo obtener muestras de biopsia, introducir stents o realizar una ablación de tejido durante una endoscopia, y puede ser relevante para el tratamiento local de infecciones fúngicas

No contamos con ninguno de estos procedimientos

Tomografía computarizada

Tomografía computarizada

Tomografía computarizada PET (PET CT)

Resonancia magnética

Resonancia magnética

Resonancia magnética PET (PET RMN)

Ecografía/Ultrasonido | Rayos X

Ecografía/Ultrasonido

Rayos x

Otro procedimiento de imagen

Otro. Indique por favor:

Endoscopias

Broncoscopia (incl. lavado broncoalveolar)

Colonoscopia

Gastroscopia

Laringoscopia

Endospcopia nasal

En promedio, ¿cuánto tiempo se tarda en obtener...

Número de días

¿Una tomografía computarizada de tórax en pacientes con sospecha de neumonía?

¿Una tomografía computarizada de la cabeza en pacientes con sospecha de infección cerebral?

¿Una resonancia magnética cerebral en pacientes con sospecha de infección cerebral?

Una exploración PET CT para descartar cualquier otro sitio infectado

Una broncoscopia con fines de diagnóstico (lavado broncoalveolar, biopsia, etc.)

¿Existe alguna política local para evitar el lavado broncoalveolar en pacientes con trombocitopenia?

No

Sí. Por favor, proporcione datos sobre el corte de recuento de plaquetas/trombocitos:

**3.1.4 Cirugía**

¿Está disponible la cirugía en su institución como parte de las estrategias de tratamiento antimicótico?

Sí

No

Desconocido

En promedio, ¿cuánto tiempo se tarda en realizar una cirugía en pacientes con una infección fúngica invasiva grave o potencialmente mortal en...

Número de días

Extracción de dispositivos permanentes u otro material extraño (p. ej., cateter port, articulaciones protésicas, stents vasculares, etc.)

Cirugía torácica

Cirugía abdominal

Cirugía de desbridamiento para otras regiones (p. ej., tejido blando profundo)

Neurocirugía (infección cerebral)

Cirugía ocular (infección orbitaria)

Cirugía de oído, nariz y garganta

Cirugía genitourinaria

Cirugía ortopédica (infección ósea/articular)
